# Qualitative research capacity building: Reflections from a UK-Kyrgyz Republic global partnership

**DOI:** 10.7189/jogh.11.03127

**Published:** 2021-12-18

**Authors:** Zainab K Yusuf, Maamed Mademilov, Gulzada Mirzalieva, Mark W Orme, Claire LA Bourne, Talant Sooronbaev, Sally J Singh, Dominic Malcolm

**Affiliations:** 1 Department of Respiratory Sciences, University of Leicester, Leicester, UK; 2 Centre for Exercise and Rehabilitation Science, NIHR Leicester Biomedical Research Centre – Respiratory, University Hospitals of Leicester NHS Trust, Leicester, UK; 3 National Centre of Cardiology and Internal Medicine (NCCIM) named after academician M. Mirrakhimov – Bishkek, Kyrgyz Republic; 4 School of Sport, Exercise and Health Sciences, Loughborough, Loughborough University, UK

Researchers have emphasised the practicalities associated with building global research partnerships, including ensuring good communication and having equitable relationships [1-3]. These challenges are complicated by cross-national inequities and perceived power dynamics, further impacted by the COVID-19 pandemic [4]. However, the existing literature on the challenges of global health partnership working largely avoids ‘the elephant in the room’; that is to say, the practicalities of doing the research itself [2,5]. Moreover, there is an implicit but false assumption that research methods are culturally neutral, when in reality the dominant modes of enquiry vary across time and space. Addressing these issues is therefore crucial to the mission of global health and the process of decentralising and democratising knowledge platforms [5-7]. Consequently, the aim of this viewpoint is to reflect on the practical and real-world challenges of combining empirical research with capacity building to inform future global health research partnerships.

## CONTEXT AND METHOD

The National Institute for Health Research (NIHR)-funded Global Health Research Group on Respiratory Rehabilitation (RECHARGE) began in 2019 and aims to address chronic respiratory diseases in low and middle-income countries (LMICs) through the development of culturally-appropriate pulmonary rehabilitation (PR) programmes. Embedded throughout the project is the goal of increasing research capacity [8], developing the skills required for LMIC researchers to conduct projects independently in the future.

This viewpoint foregrounds the perspectives of two respiratory physicians and researchers from the Kyrgyz Republic (MM & GM) who previously had minimal experience of conducting qualitative research, and two of the UK-based team who specialise in qualitative methods (ZY & DM). The specific focus of this part of the Kyrgyz RECHARGE project was to explore the respective perspectives of adults living with post-TB lung disease and health care professionals involved in treatment.

The UK team’s reflections on capacity building began in June 2020 but accelerated when MM was awarded second prize in a competition for young scientists by the Association of Internal Medicine Physicians of the Kyrgyz Republic in March 2021. First, ZY and DM consolidated those reflections in a series of written questions. MM and GM’s written responses were then discussed during a recorded Microsoft Teams meeting in September 2021. ZY and DM combined the written and audio material to create the narrative presented. MM and GM edited the first draft to ensure their views were faithfully represented and the manuscript was then reviewed by principal investigators of the RECHARGE project and others involved in qualitative components. The main themes to emerge reflected on: the cultural specificity of methods and partnership building through research; the value of probing; data analysis; and implications for future practice.

## METHOD AS CULTURALLY SPECIFIC

MM and GM were keen to emphasise that ‘qualitative research is not popular in post-Soviet countries’ and that ‘nobody really knows how to practice this type of research’. Compared to the West, qualitative research in Central Asia is regarded as a ‘new’ and ‘unique’ method, with few published studies in clinical research. Consequently, MM noted, ‘it is difficult for many to accept and understand the essence of these [qualitative] studies’.

## PARTNERSHIP BUILDING THROUGH RESEARCH

MM and GM reported being apprehensive when they were chosen by TS in 2019 to lead the qualitative research. Their assigned tasks included co-devising interview guides, conducting interviews and focus group (FG) discussions, using thematic analysis [9], and authoring conference papers [10] and journal submissions (currently under review). MM and GM acknowledged ‘fear of missing out on [any important interview data]’ due to the novelty (for them) of the inductive principles of this qualitative approach’.

The UK team sensed this uneasiness during the weekly meetings held in 2019 to support the project’s development. Subsequently MM and GM noted that these ‘joint discussions…provided helpful advice’. Additionally, MM noted that ‘confidence comes with each new interview, trying to understand the research question, delving into each topic and noticing details that might have otherwise been missed’. The team engaged in a process of continuous verbal and written feedback and ‘feed forward’, fostering open discussion by augmenting scheduled meetings with less formal communication, eg, via WhatsApp messages and calls. These helped to support both the empirical research and skills development, each of which should be seen as iterative processes [11].

**Figure:**
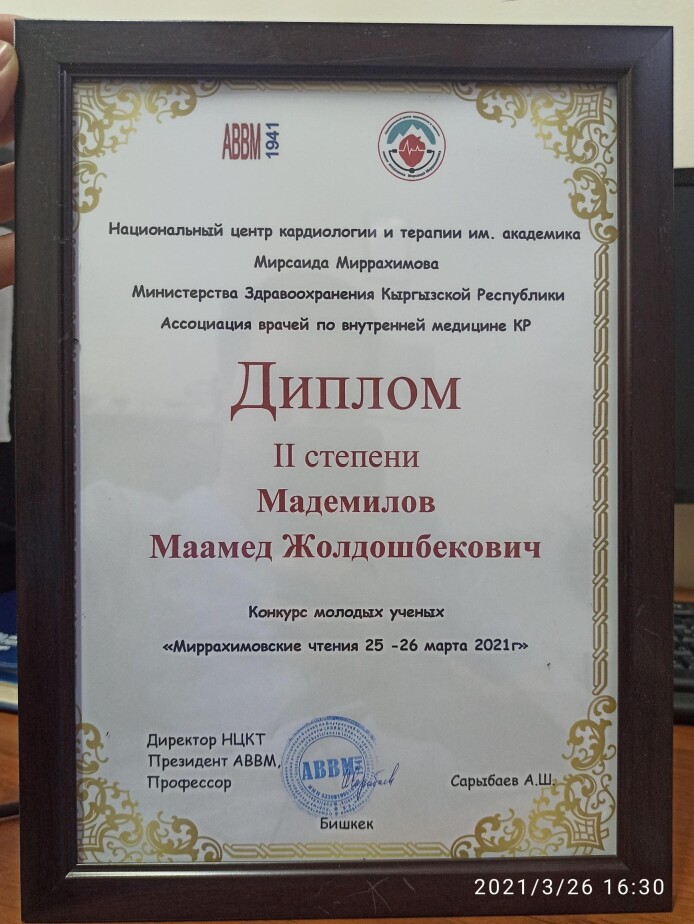
Photo: The text in the photo is in Russian and translates to ‘Second prize awarded to Dr Maamed Mademilov at the ‘Association of Internal Medicine Physicians of the Kyrgyz Republic Conference for young scientists’ (from Global RECHARGE, used with permission).

## THE BREAKTHROUGH: SEEING THE VALUE OF PROBING

The UK team noted that the initial interview transcripts showed little evidence of probing (eg, teasing out meaning, or seeking clarification). MM and GM were encouraged to critically reflect on their interviewing experiences. This was a particular challenge for MM and GM who, after completing three interviews, suggested taking some time out to transcribe the data and make plans for subsequent interviews.

A breakthrough occurred when, during the process of data familiarization, some significant yet unanticipated findings emerged. While the manifestation of stigma was predicted, MM and GM were surprised by the degree of impact on patients. The team decided to probe this further. A challenge for MM and GM was that the difference between ‘probing’ and ‘leading’ questions seemed indistinct, but they recognized that the development of an appropriate PR programme required a deeper exploration of stigma than interviewees would initially volunteer. They saw that interviewing required flexibility, and that exploratory questions were both permissible and desirable within the logic of qualitative research. MM encapsulated this through an analogy; ‘During archaeological excavations, after finding one coin, you can find a whole city’. The results of probing this topic demonstrated the distinct contribution of qualitative research.

Probing was an ongoing part of discussions because the challenge of explaining the difference between ‘probing’ and ‘leading’ raised a number of paradoxes for even the more experienced qualitative researchers. While the UK team were conscious of the need to make the Kyrgyz researchers be aware of and limit how their assumptions affected the data, they ascribed to an approach premised on the importance of recognizing a multiplicity of viewpoints. A structured interview schedule had been co-created to enable MM and GM’s data gathering, but the UK team were now emphasizing the need to remain flexible and explore the ‘gaps’ that had not been anticipated. This challenge required the UK team to emphasize that the authoritative position they had assumed as the project's qualitative ‘experts’ was not immutable; that MM and GM should lead on certain aspects of decision-making.

## DATA ANALYSIS: ‘THE MOST DIFFICULT PART’

Another key challenge was data analysis; specifically, how to conduct an objective analysis when, as MM noted, there could be ‘one thousand opinions about one thing’. First, we embarked on a regular cross-checking of interpretation to provide a form of triangulation. Second, we acknowledged the time-consuming nature of qualitative data analysis – ‘hard, painstaking work that requires constant evaluation’ (MM) – additional time was factored in for this stage of the process.

## IMPLICATIONS FOR FUTURE PRACTICE: AN ADDED BONUS

MM and GM are more confident as they embark on the project’s next phase of qualitative data gathering. They have shared their findings and experiences at national forums, congresses and other meetings with doctors, researchers and students in Central Asia, generating wider publicity. They hope to teach other physicians in the Kyrgyz Republic and other post-Soviet countries about qualitative research. For MM, ‘qualitative research is a very interesting, exciting process that opens up new horizons, data that could be hidden or not available to the eyes of many researchers.’ This is key evidence of success from a capacity building perspective [5].

Additionally, and while not a specific project goal, the experience of conducting qualitative research has informed MM and GM’s clinical practice and led them to implement a more sensitive, patient-centered approach to care. Clinical consultations for MM and GM now involve ‘asking open-ended questions, paying more attention to routine aspects of patients’ daily lives, and searching for connections’.

## CONCLUSION

Capacity building should be embedded in all global health projects and considered at least equally important to the research itself [8]. Qualitative research is an iterative learning process which requires practice, periods of reflection, and can be facilitated through ongoing discussions. To be faithful to the principles of a qualitative approach, the process must be more of an exchange of ideas than instruction and direction. This in turn will build stronger partnerships and create a feedback loop where co-researchers embrace each other’s positions. In this respect, qualitative research has much in common with the philosophical and social justice principles of decolonization [5].

While learning to craft a partnership based on trust and respect has previously been addressed by research [2,3], a crucial lesson from this project was that these qualities flow from the emotional satisfaction of learning a new research skill, applying it effectively, advancing the project, and learning how to transfer these skills. Consequently, Abimbola and Pai’s claim that ‘you cannot truly help or support people, be their allies and enablers, without seeing the world through their eyes and seeing yourself as they see you’ [5] is as true in relation to co-researchers as it is to research participants.
